# Hardware-Mappable Cellular Neural Networks for Distributed Wavefront Detection in Next-Generation Cardiac Implants

**DOI:** 10.1002/aisy.202200032

**Published:** 2022-05-12

**Authors:** Zhuolin Yang, Lei Zhang, Kedar Aras, Igor R. Efimov, Gina C. Adam

**Affiliations:** Department of Electrical and Computer Engineering, The George Washington University, Washington, DC 20052, USA; Department of Electrical and Computer Engineering, The George Washington University, Washington, DC 20052, USA; Department of Biomedical Engineering, The George Washington University, Washington, DC 20052, USA; Department of Biomedical Engineering, The George Washington University, Washington, DC 20052, USA; Department of Electrical and Computer Engineering, The George Washington University, Washington, DC 20052, USA

**Keywords:** arrhythmia, cardiac implants, cellular neural networks, distributed computing, machine learning, memristors, resistive random access memory, wavefronts

## Abstract

Artificial intelligence algorithms are being adopted to analyze medical data, promising faster interpretation to support doctors’ diagnostics. The next frontier is to bring these powerful algorithms to implantable medical devices. Herein, a closed-loop solution is proposed, where a cellular neural network is used to detect abnormal wavefronts and wavebrakes in cardiac signals recorded in human tissue is trained to achieve >96% accuracy, >92% precision, >99% specificity, and >93% sensitivity, when floating point precision weights are assumed. Unfortunately, the current hardware technologies for floating point precision are too bulky or energy intensive for compact standalone applications in medical implants. Emerging device technologies, such as memristors, can provide the compact and energy-efficient hardware fabric to support these efforts and can be reliably embedded with existing sensor and actuator platforms in implantable devices. A distributed design that considers the hardware limitations in terms of overhead and limited bit precision is also discussed. The proposed distributed solution can be easily adapted to other medical technologies that require compact and efficient computing, like wearable devices and lab-on-chip platforms.

## 1. Introduction

Machine learning (ML) algorithms are being adopted to analyze medical data in specialties like radiology, oncology, and cardiology, promising faster interpretation with accuracy close to doctors’ diagnostics.^[1]^ The next frontier in computing technology is to bring these powerful algorithms to implantable medical devices, which requires automation of real-time life-saving therapeutic decisions without the physician’s presence. An example is the need for improved medical solutions for life-saving cardiac defibrillation therapies, that can detect bioelectric anomalies (e.g., cardiac arrhythmias) and act on this data locally for real-time therapy delivered within tens of seconds or minutes since the onset of life-threatening ventricular fibrillation (VF). The statistics put this challenging technological need in perspective: ventricular arrhythmias such as VF are responsible for over 700 000 sudden cardiac deaths a year in the USA and Europe.^[2]^ VF is a common, life-threatening arrhythmia characterized by chaotic asynchronous electrical activity of the cardiac muscle, which results in death within 10 minutes.

Individual differences in physiological mechanisms, anatomic and genetic determinants, and etiologies of various arrhythmias impact the course of treatment. Ablation therapy, while promising, remains a work in progress. Therefore, on average, defibrillation therapy delivered by implantable cardioverter defibrillators (ICDs) remains the most effective treatment as antiarrhythmic drugs have limited efficacy and can be associated with adverse side effects. Implants have to be biocompatible, organ conformal, and small enough to minimize the tissue damage and be capable of independent autonomous operation without external intervention. Low power is an essential characteristic to avoid the heat damage to the tissue and prolong the lifetime of the embedded battery for many years without recharging.^[3]^ Currently, most volume of the ICD has been occupied by batteries, which has limited the volume reduction and the computing capacity. ICD has local computing based on a microprocessor to detect and differentiate arrhythmia to offer different treatments, but the resolution provided by ICD is really low typically limited to only one or a couple of sensors; as such, the ability to detect arrhythmia wavefronts is non-existent. The data can be read wirelessly by the physician during periodic checkups. Increasing the sensing resolution is desired but the local computing capacity has to also be increased which is difficult due to power constraints. Wireless data transmission for processing of data outside of the body is not a viable solution either, as real-time data transfer between the implant and the external world requires a significant amount of power, even increasing the volume of the implant while introducing delays and additional security risks. That is why these systems have focused mostly on local real-time signal processing.

However, due to the low resolution for sensing and therapy, high-energy biphasic shocks are needed to effectively terminate life-threatening high-frequency arrhythmias. These high-energy shocks can lead to myocardial damage and associated comorbidities and it is a painful and traumatic experience for patients, especially when delivered inappropriately when arrhythmia is not present due to poor sensing. On the other hand, multipulse therapy (MPT) utilizes well-timed trains of low-energy electric pulses. Experiments on animal and human heart tissue showed that when appropriately timed, MPT significantly decreases the high-energy defibrillation threshold by almost an order of magnitude. Moreover, recent first-in-human clinical trial demonstrated safety and efficacy of MPT in patients with atrial fibrillation,^[4]^ which is not possible with current high-energy ICDs due to pain and discomfort caused by high-energy shocks. However, as it is administered also through transvenous leads, the issue of low resolution remains.

To study the mechanisms of arrhythmias and develop suitable MPT for clinical use, high-definition electrically or optically mapped electrocardiograms (ECG) data must be used, which requires a large number of sensors to map the cardiac tissue surface. High-definition ventricular arrhythmia sensing integrated with electrotherapy is an emerging concept enabled by organ-conformal electronics platforms. Prototype organ-conformal electronic platforms have been developed with noncontact sensors and actuators and tested in vivo^[5]^ but have limited resolution. Increasing the density of sensors and actuators is underway,^[6]^ promising a personalized electrotherapy solution to terminate life-threatening tachycardias with two orders of magnitude less energy than a typical shock.^[7]^ Such platforms could be used to predict fibrillatory wavefronts and enable their prevention using high-definition sensing and ultralow-energy electrotherapy that does not cause pain and discomfort.

The high definition is a critical requirement as multiple rotors can be simultaneously present in the myocardium^[8]^ during an arrhythmia event and generate the seemingly chaotic pattern on the electrocardiogram that is the hallmark of atrial and ventricular fibrillation. The ventricular fibrillation rotors can be identified based on individual wavefronts, and wavebreaks are represented by phase singularities. The wavefront is defined as isolines of the phase that terminate either at boundaries or at singular points with the phase field (phase singularities^[9]^). Although the exact data resolution needed to extract these chaotic wavefronts is still under investigation, we estimated that >10 000 sensors, sampled at ≈500 Hz with 12-bit digitization, can produce an accurate map for the entire human heart. Such a system would produce >60 MB s^−1^ of data which must be processed in milliseconds, an insurmountable task for serial computation, especially on microprocessors of miniature implantable devices with limited energy resources. Real-time smart and energy-efficient computation is needed to process the data and trigger the local activation of actuators. To our knowledge, no organ conformal electronics platform has embedded computing for local data interpretation and millisecond decision-making, as needed for real-time life-saving therapy such as arrhythmia electrotherapy.

In this work, we propose the use of distributed computing neural network algorithms which are hardware mappable, to provide high classification sensitivity, specificity, accuracy, and precision in determining the challenging spatiotemporal dynamics of cardiac electrical signals. Artificial neural networks can process a large amount of data in a parallel fashion and “learn” its patterns. As their name suggests, artificial neural networks are inspired by biological brain and can provide intelligent computing solutions. Deep learning techniques, such as convolutional neural networks, have been demonstrated to perform with >93% accuracy for the classification of ECG heartbeats.^[10–12]^ These complex networks can be used for classification of heartbeat by heartbeat of data obtained from bedside ECG recording equipment, but they have yet to be applied in current low-resolution ICDs that shock the entire heart due to computational complexities and limited microprocessor capabilities.^[13]^ However, for new types of high-definition organ-conformal platforms, they are impractical to physically realize due to their complexity for a large number of recording channels and also unsuitably centralized for the spatiotemporal tracking of wavefronts and wavebreaks as needed for precise therapy by distributed electric field. To our knowledge, no neural network algorithm has been proposed for the identification of wavefronts.

**Figure 1.**
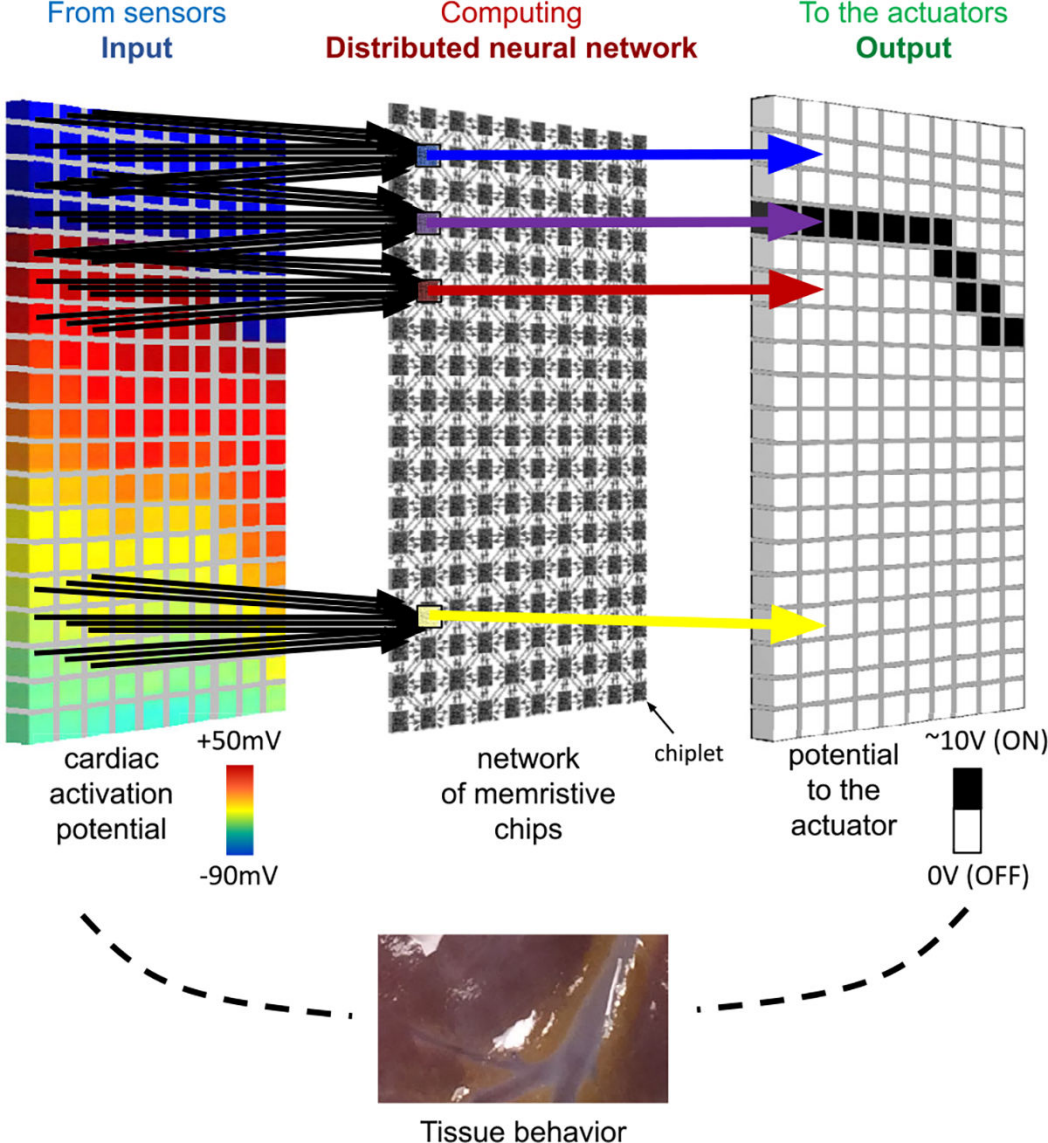
Distributed computing for electrical wavefront determination: Proposed technology using integrated network of sensors, computing chiplets distributed in a cellular neural network architecture, and actuators that will allow high-definition mapping, interpretation, and therapeutic response in a closed-loop fashion.

This work describes a distributed computing algorithm based on cellular neural networks that is readily mappable to memristor-based hardware circuitry and could enable a closed-loop solution that includes spatially distributed sensing, data processing, and any required actuation for therapy (Figure 1). The cellular neural network maps well to a spatiotemporally distributed architecture and would enable a high-speed high-data-throughput computing solution. Any other type of neural network, for example, a multilayer perceptron or a convolutional neural network, would require hardware implementation in a single chip which would have to be connected to a multitude of sensors and actuators, with density limitations due to the interconnects. This proposed cellular neural network architecture was chosen as most suitable because it takes advantage of its natural tiled organization to easily map it to a distributed network of identical computing chiplets, as shown in Figure 1. We consider a chiplet to be a small integrated circuit (IC) of submillimeter dimensions that contains a well-defined subset of functionality and is designed to be combined with other chiplets in the organ-conformal platform. Each chiplet implements a cell unit of the cellular neural network, processing only local sensor information from itself and its neighbors and providing an output only to its local actuator.

The size, area, and power constraints are particularly important for this application. Emerging computing technologies, like memristor crossbars,^[14]^ have significant potential in the More-than-Moore era, promising orders of magnitude better energy efficiency and compact implementation^[15,16]^ of use in novel computing systems for implantable devices. A memristor commonly uses metal/insulator/metal sandwich structures, which include two layers of electrodes and an intermediate layer of memristive functional material, which is called the insulator.^[17,18]^ Memristor devices can be fabricated as small as 2 nm, and^[19]^ their resistance transition characteristics are closely associated with their electrodes and the switching materials. The device needs “forming” to create filamentary path(s) in the insulator and then reversibly set and reset to program the device to a desired conductance state between low (OFF) and high (ON) states. Thanks to its ionic transport, the programmed state is retained without static power dissipation. Memristor devices can be integrated with complementary metal–oxide–semiconductor (CMOS) control circuitry as dense matrices (crossbars) of artificial synapses to implement vector matrix multiplication using Ohm’s law,^[14,20–22]^ which is a fundamental operation in neural networks. This behavior enables a natural solution for the implementation of templates for the proposed cellular neural network computing, to be integrated directly with sensors and actuators. This approach allows for flexibility, requiring the design of only one chiplet and its tape-out in as many samples as needed for the size of the network at hand. The proposed solution can be used to develop the next-generation implantable devices that can provide low-energy therapy, thanks to high-resolution sensing, local computing, and precise actuation.

The remainder of the article is organized as follows. Section 2 describes the methodological details, the data obtained from human cardiac tissue, as well as the algorithm and the performance metrics used. Section 3 introduces the evaluation of the proposed methodology on the dataset, considering the optimization of hyperparameters such as the learning rate, weight initialization, binarization, as well as the impact of noise and quantization in the input and templates on the inference results. Section 4 concludes with a discussion of the algorithmic results and their potential mapping to a memristor-based hardware implementation.

## 2. Experimental Section

### 2.1. Data Gathering and Preprocessing

This study utilized representative data obtained from a deidentified donor human heart from the Washington Regional Transplant Community (Church Falls, VA). The study was approved by the Institutional Review Board at the George Washington University.

**Figure 2.**
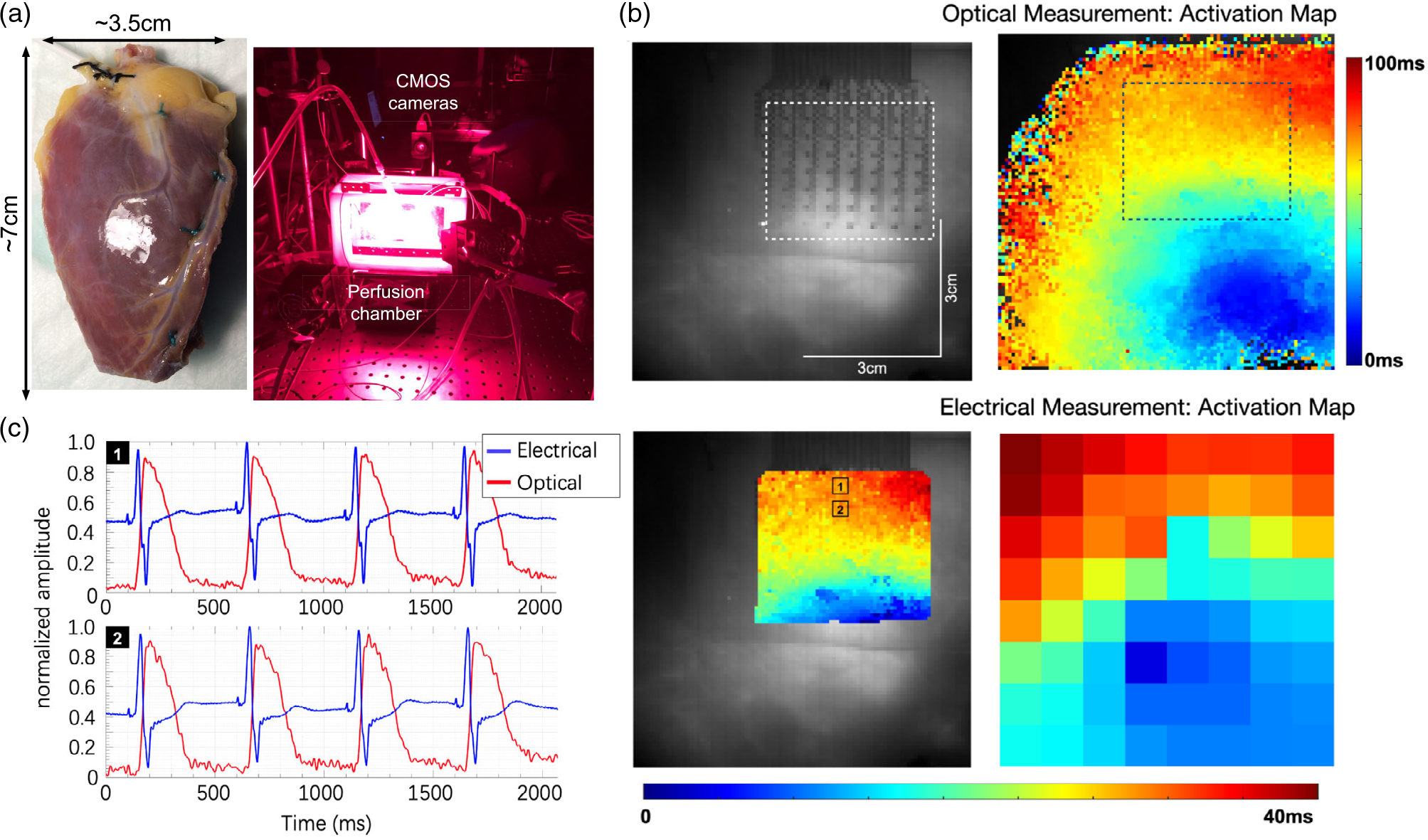
Data gathering. a) Human left ventricular tissue wedge and experimental setup. b) Simultaneous optical and electrical cardiac mapping. c) Corresponding representative electrical and optical signals. Figure 2b,c are reproduced with permission.^[24]^ Copyright 2022, American Heart Association.

The experimental apparatus and procedures are explained in detail in the study by Aras et al.^[23]^ Briefly, the ventricular tissue was prepared as a wedge with average dimension of 7 cm × 3.5 cm (Figure 2a). The tissue was then mounted in a temperature-controlled, pressure-controlled, and an oxygenated optical mapping setup (Figure 2a). Optical action potentials were mapped from ≈7 cm × 7 cm field of view using a MiCAM05 (SciMedia, CA) CMOS camera (100 × 100 pixels) and sampled at 1 KHz sampling rate.

The dataset consisted of 1000 optical mapping images of the epicardium tissue recorded at 1 kHz sampling rate with a size of 100 × 50 pixels. 800 images were used for training and 200 for testing. The dataset included complete recordings of several fibrillation events, enabling the analysis of various wavefront patterns during fibrillation as part of this work. Optical recordings were used because they provide higher-resolution mapping than flexible electrode arrays. However, these results were directly applicable to electrically recorded data, as shown in Figure 2b,c.^[24]^ Studies into the resolution required to extract any possible chaotic rotors in human tissue are still under investigation and higher-resolution setups are being developed.

Analysis in the phase domain was typically done for such studies, as the wavefront propagation and the singularities could be easily detected in the phase domain. The time domain optical raw data recorded by the cameras was preprocessed to transform it into the phase domain with a scale between −*π* and *π* through the Hilbert transform.^[25]^ The Hilbert transform is an efficient signal analysis method for nonstationary time series, especially in determining the instantaneous frequency of time-varying signals, such as ventricular arrhythmias. Detection of these subtle frequency changes and potentially recognizing the initiation and/or termination of VT/VF is very important in understanding the mechanisms of arrhythmia. Given a real-time function *x*(*t*), its Hilbert transform was defined as^[26]^

(1) $$\hat{x}(t)=H[x(t)]=x(t)*\frac{1}{\pi t}=\frac{1}{\pi }\int_{-\infty }^{+\infty }\frac{x(\tau )}{t-\tau }\text{d}\tau$$

**Figure 3.**
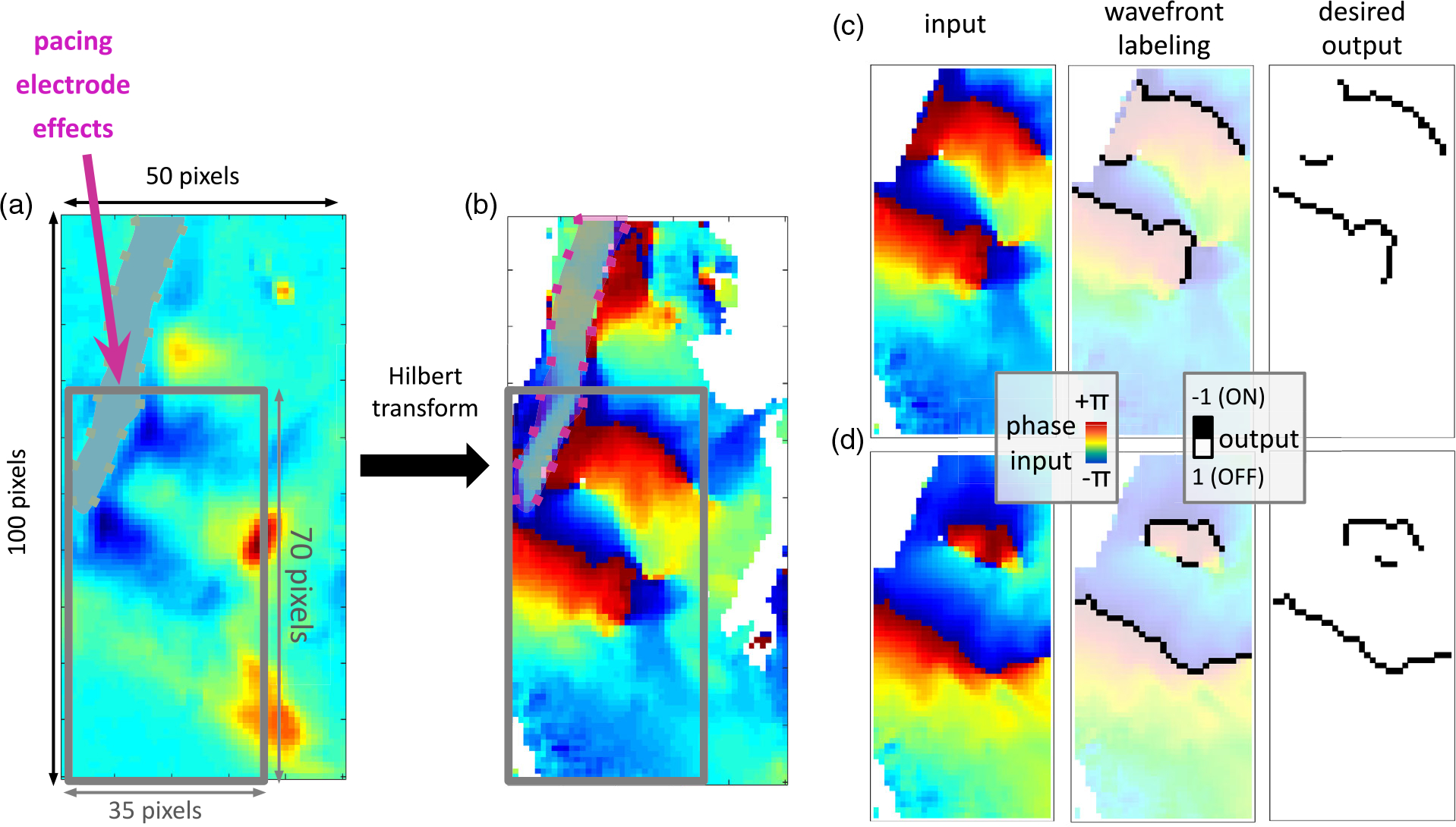
Data preprocessing. a) Example of raw optical phase map (100 × 50 pixels) recorded during VF in the human heart preparation showing the influence of the pacing electrode on the obtained signal. b) Example of Hilbert-transformed optical phase map. A subset (70 × 35 pixels) was selected to avoid network confusion due to pacing electrode effects. c) Example of input data used for training and its labeling. d) Example of input data used for testing and its labeling.

Figure 3a shows a raw optical signal and Figure 3b shows its phase-domain equivalent that was further preprocessed before looking at the wavefront. More details are presented in prior work.^[23]^ A wavefront was located at the edge of phase ∅(*t*) = *π* (red) and phase ∅(*t*) = −*π* (blue) on the blue side. The wavefronts were labeled manually because the noise and the undesirable artifacts of the pacing electrode might affect the precision of the labels and affect the training results afterwards. For each data sample, a corresponding phase map 3b and its wavefront mapping 3c served as input and desired output, respectively, for the neural network core.

Due to the unavoidable interrupts during the hour-long experiments and the underlying condition of the available human heart tissue, noise was an inevitable occurrence in the dataset. Noise is regarded as the irregular small section of pixels rapidly changing in the range from *π* to −*π*, as well as the value of pixel remaining constant throughout the measurement. The pacing electrode could also introduce significant artifacts due to its large size, needed to provide mechanical robustness during insertion into the rather stiff human cardiac muscle tissue. To avoid these unwanted effects, the data was cropped to 70 × 35 pixels and the pixels containing the pacing electrode were removed, as shown in 3b vs. 3c.

### 2.2. Cellular Neural Networks

Given the tight requirements for high speed and low-power hardware, the cellular neural network is a promising topology for distributed computing, based on a fixed number of interconnected processing units called “cells.” Each unit, for example, unit *ij* at row *i* and column *j*, could be implemented by a computing chiplet, processing only local information from itself and its neighbors, with small size and energy requirements. The inputs *u*_*ij*_(*t*) at time *t* were fed into the network and outputs *y*_*ij*_(*t*) were obtained. The output of a processing cell *ij* was determined by the state of the cell *x*_*ij*_(*t*) according to Equation (2).


(2) $$y_{ij}(t)=\left(\left|x_{ij}(t)+1\right|-\left|x_{ij}(t)-1\right|\right)$$

The state of cell *ij* at time *t* was calculated using the differential equation (3) taking into consideration all the cells in the neighborhood of size *M*× *N*. This work included only the nearest neighbors (neighborhood size = 3 × 3) to keep the results mappable to a potential compact hardware implementation. However, the neighborhood could include further away neighbors, for example, a neighborhood of size 7 × 7 included one central cell and 48 neighbors.


(3) $$\frac{dx_{ij}(t)}{dt}=-x_{ij}(t)+\sum_{\begin{array}{l} 1\leq i\leq M\\ 1\leq j\leq N \end{array}}a_{mn}y_{mn}(t)+\sum_{\begin{array}{l} 1\leq i\leq M\\ 1\leq j\leq N \end{array}}b_{mn}u_{mn}(t)+I$$

In Equation (3), the inputs *u*_*mn*_ and outputs *y*_*mn*_ of its cell and neighboring cells were weighted via the matrix elements *a*_*mn*_ and *b*_*mn*_ of two matrices A and B of size *M* and *N*. The matrix A linked the outputs *y*_*mn*_ to the state *x* via its elements *a*_*mn*_, while template B similarly linked the inputs *u*_*mn*_ to the state *x*, respectively. These matrices were called templates and were used repeatedly for each cell. Training the network means determining the values of templates A and B and of bias *I*.

Several algorithms were used for training these networks, including, random weights change,^[27]^ Kalman filters,^[28]^ genetic algorithms,^[29]^ and backpropagation.^[30]^ The random weight change^[27]^ is a hardware-friendly algorithm for on-chip training on a wide range of tasks, but it involves large number of training epochs to obtain accurate templates. Kalman filters have been used to obtain accurate output from the inaccurate input information, minimizing the mean of squared error by estimating the inner states of any dynamic process.^[30]^ Genetic algorithms have been shown to train the network with desirable accuracy and robustness, but the evaluation of the fitness functions is computationally very expensive.^[29]^

We have defined a training algorithm based on backpropagation and batch updates robust to template nonidealities. Following initialization, the network will calculate the corresponding error for each image in the batch. The templates A, B, and bias *I* will be updated after each batch calculation. The process was repeated for all images in the training dataset to minimize the error between the obtained wavefront map output and the desired output. The network took several epochs to converge and several performance metrics, as shown in the next section, could be used to track the convergence.

For the case of the typical adapted stochastic gradient descent backpropagation training algorithm, the error was calculated based on

(4) $$e_{ij}[k]=\frac{1}{2}\left(d_{ij}-y_{ij}^{*}[k]\right)$$

where $y_{ij}^{*}[k]$ is the output calculated by the algorithm at iteration *k* and *d*_*ij*_ is the desired cell output according to the image label. The templates *A*, *B*, and bias *I* are updated based on

(5) $$a_{mn}[k+1]=a_{mn}[k]+\eta \Delta a_{mn}[k]$$

(6) $$b_{mn}[k+1]=b_{mn}[k]+\eta \Delta b_{mn}[k]$$

(7) I[k+1]=I[k]+ηΔI[k]

with the updates of *Δ* weights

(8) $$\Delta a_{mn}[k]=\frac{1}{MN}\sum_{\begin{array}{l} 1\leq i\leq M\\ 1\leq j\leq N \end{array}}e_{ij}[k]y_{i+m-2,j+n-2}^{*}[k]$$

(9) $$\Delta b_{mn}[k]=\frac{1}{MN}\sum_{\begin{array}{l} 1\leq i\leq M\\ 1\leq j\leq N \end{array}}e_{ij}[k]u_{i+m-2,j+n-2}[k]$$

(10) $$\Delta I[k]=\frac{1}{MN}\sum_{\begin{array}{c} 1\leq i\leq M\\ 1\leq j\leq N \end{array}}e_{ij}[k]$$

where *m* and *n* are the row and column indices, respectively, of the templates *A* and *B*. *η* is the learning rate, typically a small number always >0, that defines the range of weight updates in each iteration. As seen in Equation (8), the update Δ*a*_*mn*_[*k*] for the feedback template *A* was calculated via the weighted sum of the error and the desired output for each cell. A similar update Δ*b*_*mn*_[*k*] was calculated for control template *B* based on the error and the respective input. The bias *I* was also updated accordingly based on the average error of each cell to increase the performance of the network.

To improve the wall-clock time, we used batch training as defined by

(11) $$\Delta A_{mn}[k]=\frac{1}{B}\sum_{B}\Delta a_{mn}[k]$$

(12) $$\Delta B_{mn}[k]=\frac{1}{B}\sum_{B}\Delta b_{mn}[k]$$

(13) $$\Delta I_{batch}[k]=\frac{1}{B}\sum_{B}\Delta I[k]$$

where *B* is the batch size for which the *Δ* template updates were averaged.

The error could be calculated on the obtained output as processed by the network, which took grayscale values between [−1,1] or on a binarized version of the output which could be either wavefront (−1) or nonwavefront (1). The obtained output could be binarized, either during training or after the training was complete by applying a threshold as defined by the following equation

(14) $$y_{ij}^{\text{b}}in=\{\begin{array}{l} 1if\;y_{ij}\geq threshold\\ -1else \end{array}$$

As we targeted hardware mappability, we also explored the impact of neighborhood size as well as limited bit precision weights. Limited precision templates were also considered using traditional routing-to-nearest method versus stochastic rounding. Stochastic rounding can be particularly useful in deep network training with low bit precision arithmetic.^[31,32]^ A real template value *a* which lies between a lower weight level (*A*_1_) and upper weight level (*A*_2_) was stochastically rounded up to *A*_2_ with probability (*a*–*A*_1_)/(*A*_*2*_–*A*_1_) and down to *A*_1_ with probability (*A*_*2*_–*a*)/(*A*_*2*_–*A*_*1*_). The algorithm details are included in the supplemental materials.

### 2.3. Performance Metrics

To provide a comprehensive assessment of the potential performance of the algorithm to human tachyarrhythmia events, several performance metrics were used in accordance with medical practice for binary classification tests. The desired output was a binary map with pixels on the wavefront(s) labeled as “positive” (or “ON” or black) totaling P pixels and all other pixels, not on the wavefront labeled as “negative” (or “OFF” or white) totaling N pixels. The obtained output after the image was classified by the network was a similar binary map. Some of the pixels on the wavefront were identified correctly (true positives), totaling TP pixels, while others were misclassified as negative (false negatives), totaling FN pixels. Similarly, some of the pixels outside the wavefront were identified correctly (true negatives), totaling TN pixels, while others were misclassified as positive (false positives), totaling FP pixels. These could be arranged in a typical 2 × 2 contingency table or a confusion matrix (Table 1).

Based on this classification, four important performance metrics, accuracy, precision, sensitivity, and specificity, are defined as follows. Accuracy provides a quantitative metrics of the overall performance of the algorithm, showing the percentage of the total number of pixels correctly identified.


(15) $$\text{accuracy}=\frac{\text{TP}\;+\;\text{TN}}{\text{TP}\;+\;\text{TN}\;+\;\text{FP}\;+\;\text{FN}}$$

Precision measures the performance of correctly identifying positive (wavefront) pixels. For the targeted application, it is highly important to have very high TP and low FP (high precision) to avoid applying unneeded pulses.


(16) $$\text{precision}=\frac{\text{TP}}{\text{TP}\;+\;\text{FP}}$$

Sensitivity measured how many of the positive (wavefront) pixels were identified as such.


(17) $$\text{sensitivity}=\frac{\text{TP}}{\text{TP}\;+\;\text{FN}}$$

Specificity measures how many of the negative (nonwavefront) pixels were identified.


(18) $$\text{specificity}=\frac{\text{TN}}{\text{TN}\;+\;\text{FP}}$$

The goal was to optimize the algorithm to achieve high values for all four performance metrics, accuracy, precision, sensitivity, and specificity simultaneously.

## 3. Results

### 3.1. Training Optimization

#### 3.1.1. Hyperparameter Optimization on Single-Image Training

Single-image training was used to do a comprehensive search in the hyperparameter space for learning rate, initialization, and binarization and understand the impacts and trade-offs on performance. The optimal learning rate was identified for different initializations by exploring a broad range from 10^−4^ to 10^4^ in logarithmic scale. The initializations are 1) zero-template matrices; 2) randomly generated values between −1 and 1; and 3) pre-defined templates for edge detection; details are described in Table 2.

**Figure 4.**
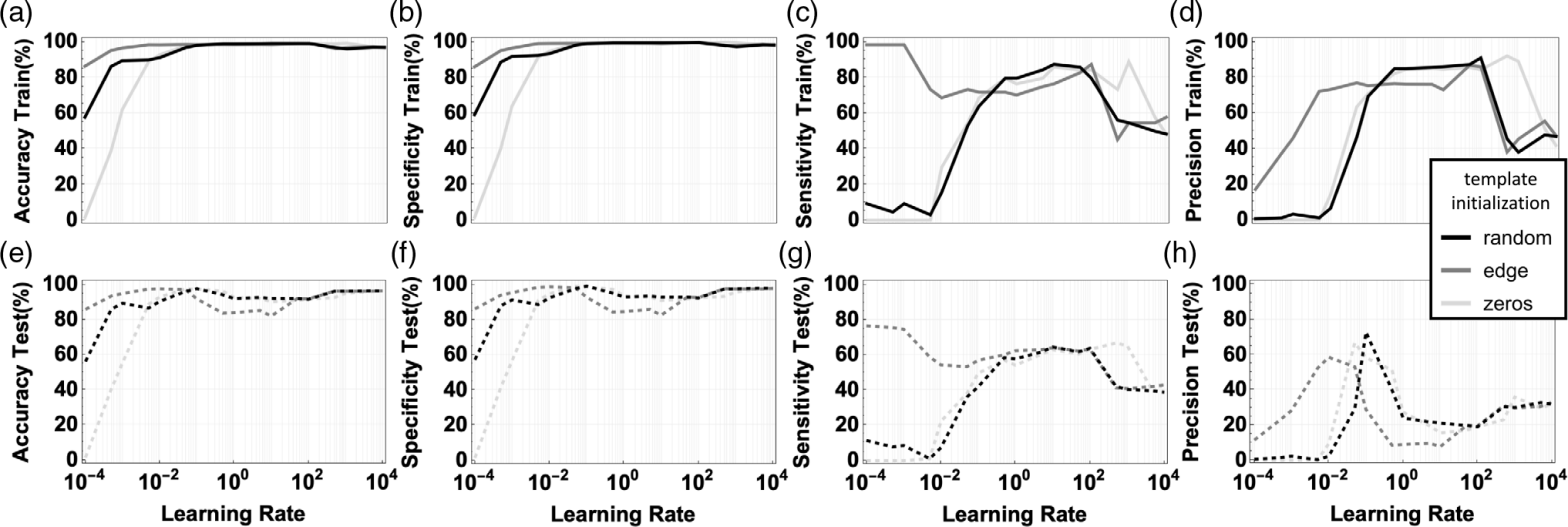
Impact of learning rate optimization and initialization optimization on training and testing performance. a,b,c,d) Evolution of sensitivity, specificity, accuracy, and precision for training and e,f,g,h) for testing, respectively, using different learning rates and different initializations. The representative image from Figure 3b and its label were used for training. Number of neighbors = 8.

As shown in Figure 4, an optimal learning rate window is visible, where all the four performance metrics are optimized. Outside of this learning rate window, the performance drops particularly for precision and sensitivity. All these metrics need to be optimized simultaneously, with precision being the most important metric to avoid false positives that would inadvertently apply unwanted pulses to the heart tissue. The highest precision is 92.16% on learning rate = 500 with all initial templates *A*, *B* and bias *I* set to 0 shown in Figure 4d. The maximum value for specificity is 99.82% at the same learning rate. The maximum value for sensitivity is 98.44% on learning rate = 0.05 with edge detection template and for accuracy is 99.38% on learning rate = 1000 with zero templates. However, it is important to note that large precision is obtained for different initializations and over a broad range of learning rates, from 0.5 to 500. As a learning rate that is too fast will result in large weight updates that can induce oscillatory behavior in the training between suboptimal local solutions, the best lower learning rate was investigated. Testing was used test the performance of these results on nine inputs. The averaged results are shown in Figure 4e–h. The highest precision is 73.08% on learning rate = 0.1 with random template initialization, while the other performance metrics at the same hyperparameters are accuracy 97.85%, specificity 99.26%, and sensitivity 42.00%.

**Figure 5.**
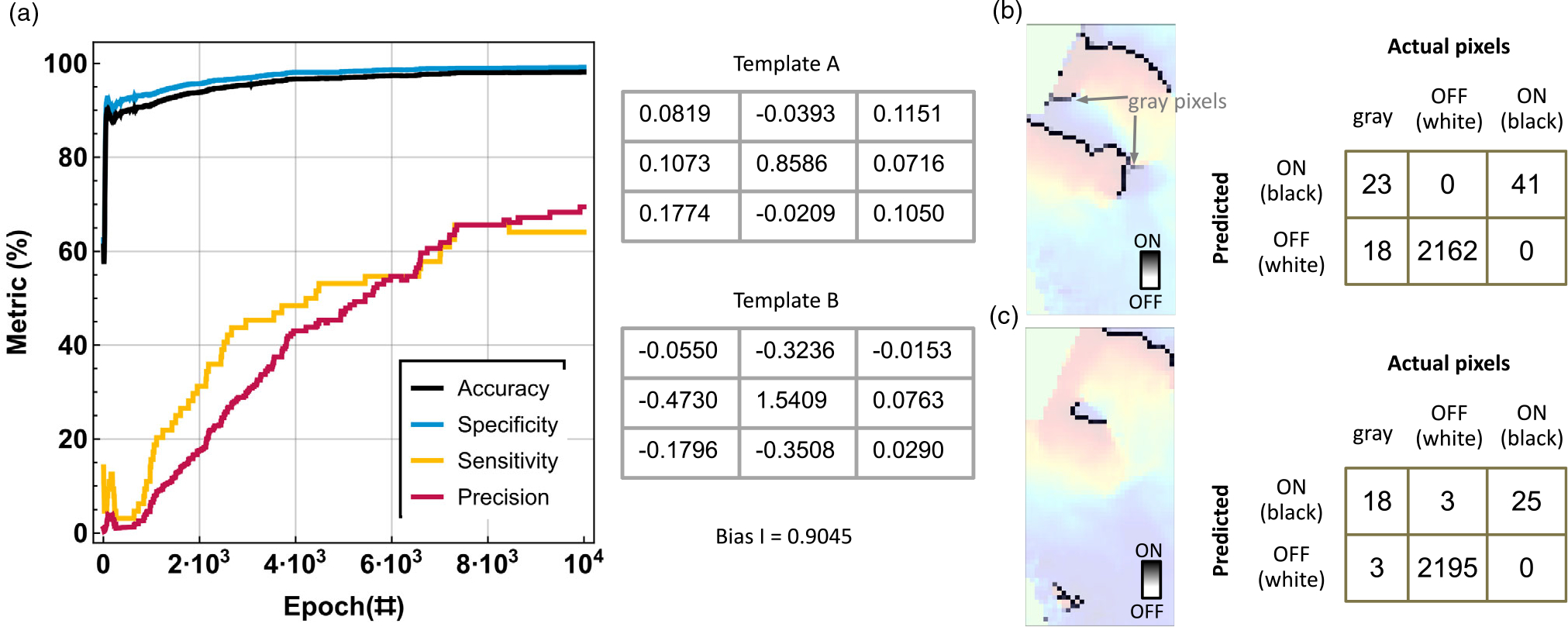
Example of wavefront identification using a trained cellular neural network with “grayscale” output. a) Evolution of the performance metrics with training with random template initialization and learning rate = 0.1. The resulting template values are included; b) output of the training image and c) example of the test image with highlighted wavefronts as determined by the algorithm and their respective performance metrics.

After evaluating the training results and testing results, within the optimal learning rate window, a learning rate of 0.1 with the random initialization of templates has shown the highest precision especially in testing results. The results in Figure 5a show the convergence curves for the different performance metrics and the obtained templates. The poor sensitivity and precision are due to the fact that 41 pixels are stuck with in-between values, as shown in Figure 5b. A test image, as shown in Figure 3c, was used for validation. The challenge with these “gray” pixels also seems to translate to the test image, as shown in Figure 5c. This method of training where the output is allowed to take “grayscale” values has a difficult time differentiating true versus false positive pixels, leading to a large number of pixels in the grayscale regime and precision of only around 70%.

**Figure 6.**
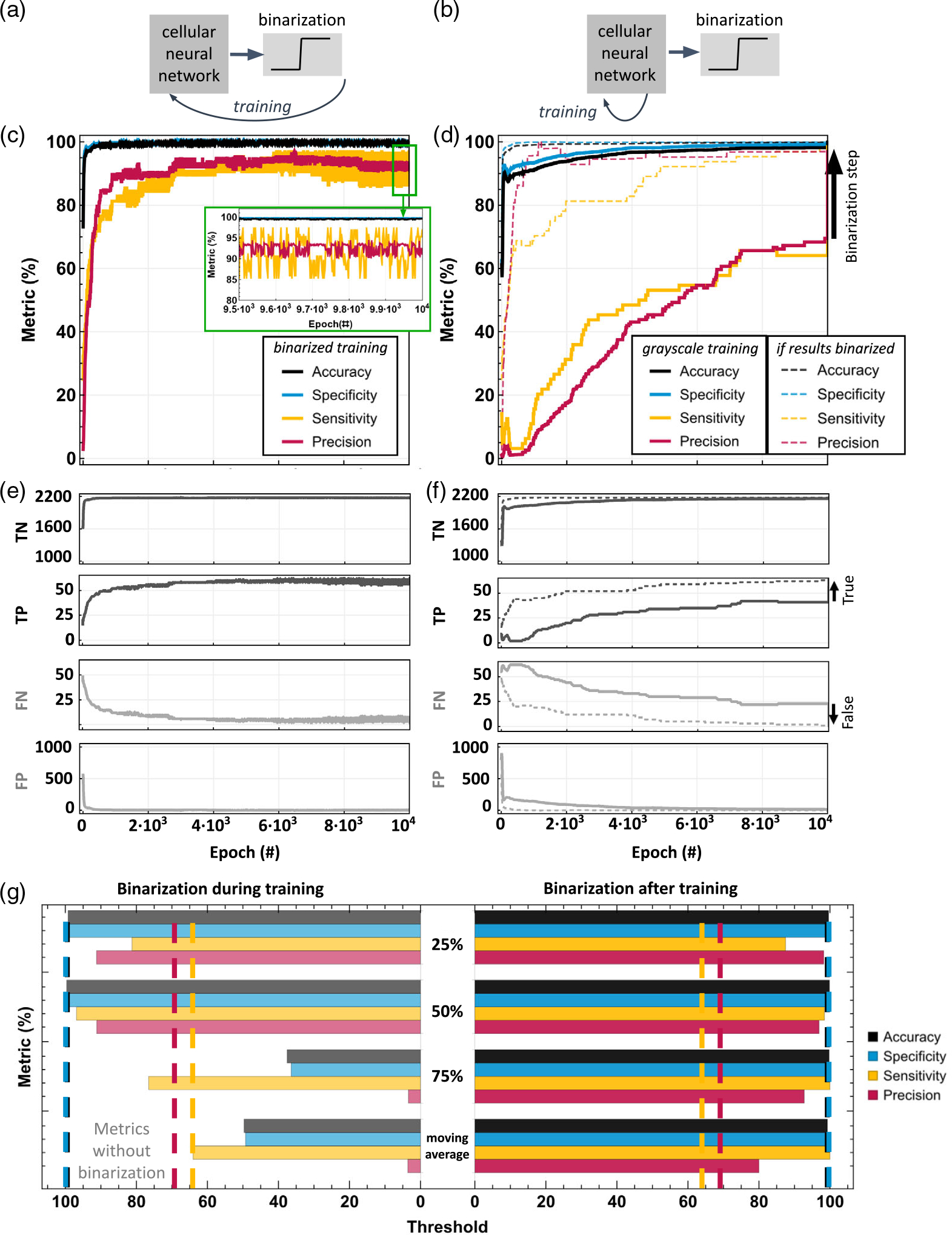
Impact of binarization on training performance. Comparison between a) binarization during training and b) binarization after training. c–d) Respective convergence curves for metrics of interest and e–f) true and falsely predicted number of pixels; g) Performance comparison using different thresholds. The dashed lines show the performance metrics of the training algorithm without binarization. The hyperparameters used for all the training results in this figure are LR = 0.1, random template initialization, and number of neighbors = 8.

These results prompt the need for a binarization approach by imposing the desired output of the network to be binary, either ON or OFF. The two methods, binarization during training and binarization after training, have been explored in Figure 6. In binarization during training approach, the output is forced to be binary after each weight update (Figure 6a). In the binarization after training approach, the network is trained by itself with output in grayscale and the output is forced to be binary once the training is complete (Figure 6b). Figure 6c vs. d shows comparatively the convergence curves for the two approaches. The binarization during training converges quickly but experiences oscillations in the metrics as the true-positive and false-negative pixels flip values and do not stay converged (Figure 6e). As the obtained output contributes further as input back into the network via the feedback template A, this creates an undesired fluctuating behavior. In particular, the sensitivity oscillates between ≈86% and 97%, while the precision oscillates between 90.7% and 93.8%. In comparison, binarization after training converges much more slowly. However, if the intermediate results were to be binarized for the purpose of visualization, the resulting curves show stable convergence behavior. The binarization after training leads to an increase in the number of true predicted pixels and a corresponding decrease in the number of false predicted pixels (Figure 6f). This method leads to accuracy and specificity above 99%, 98.44% sensitivity, and 96.92% precision for training results.

These results have been obtained with a binarization threshold of 50% midway in the output grayscale range. However, the threshold used for binarization of gray pixels can influence the performance. For example a 25% threshold favors transforming the gray pixels to black pixels, thus increasing precision and reducing sensitivity, while 75% threshold favors higher sensitivity and lower precision, as visible in Figure 6g in the binarization after training results. The threshold can also be dynamic, for example, the moving average value of the overall output. The comparison results show that binarization after training with a 50% threshold seems to achieve the most optimal solution as a trade-off between satisfactory sensitivity and precision. This method shows a significant improvement in sensitivity and precision of ≈30 percentage points over the algorithm without binarization for our proposed application. Future work can explore if this result is transferable to other applications beyond the proposed cardiac data analysis.

#### 3.1.2. Batch Training Optimization

**Figure 7.**
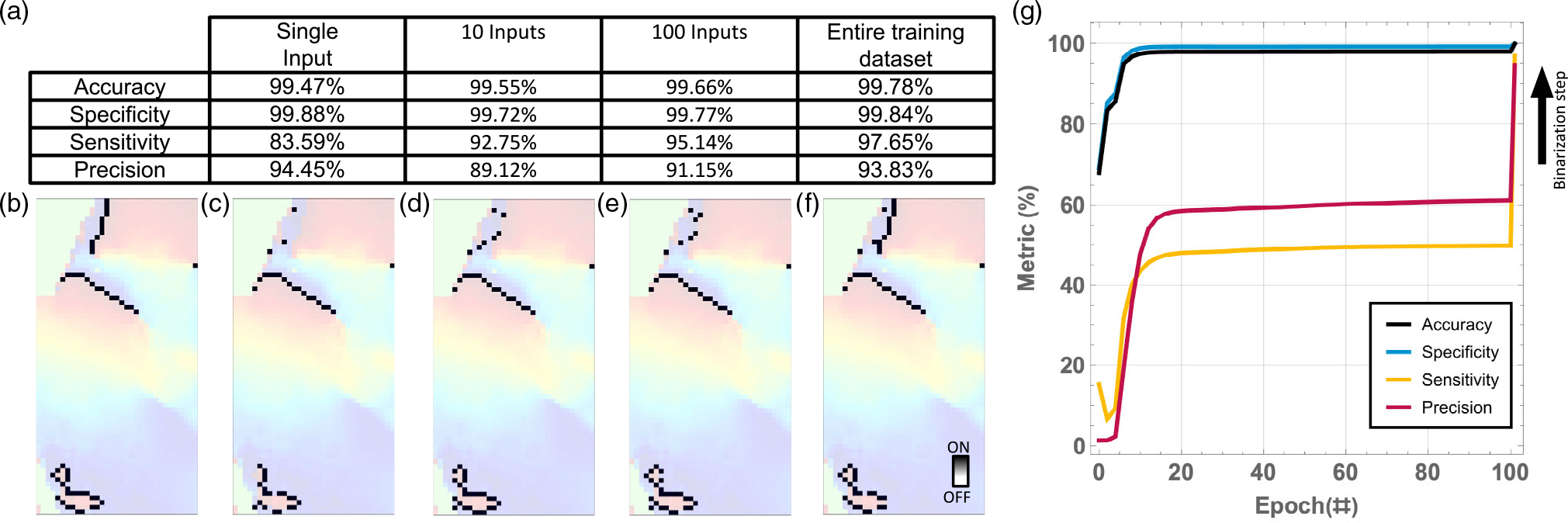
Test metrics versus size of training dataset. a) Average sensitivity, specificity, accuracy, and precision on the testing dataset; b) example of test image highlighted with desired wavefront (label); c–f) determined wavefronts using templates obtained after training on single image versus 10 images versus 100 images versus the 800 images in the training dataset. g) Convergence curves when the entire training dataset is used. The hyperparameters used for all the training results in this figure are LR = 0.1, random template initialization, number of neighbors = 8, and binarization after training. 200 images were used for testing.

The proposed system has to perform well as an inference accelerator, train off-line, and the template weights transferred a priori for inference during operation. While training on a single input is fast, the test results highlight that the obtain templates lead to poor sensitivity when applied to the 200 test images. The solution to improve the inference performance on the test dataset is batch training, as shown in Figure 7. The testing performances for training on the entire available dataset versus on subsets are compared. In particular, the test sensitivity improves as the number of samples in the training dataset increases, from 83.49% for single-input training to above 97% when 800 images are used for training. The different wavefronts, typical in ventricular fibrillation in humans, can therefore be correctly identified, as seen in an example test image in Figure 7f, versus the desired label shown in Figure 7b. As expected with the increased number of images in the dataset, the number of epochs needed for convergence decreases. Figure 7g shows training convergence for a training dataset of 800 images. The obtained training versus test metrics are very similar: accuracy 99.78% versus 99.78%, specificity 99.87% versus 99.84%, sensitivity 96.87% versus 97.65%, and precision 94.46% versus 93.83%. Future work will focus on increasing the size of the dataset with labeled optical mapping images for different cardiac tissue.

#### 3.1.3. Limited Precision Hardware-Aware Training

**Figure 8.**
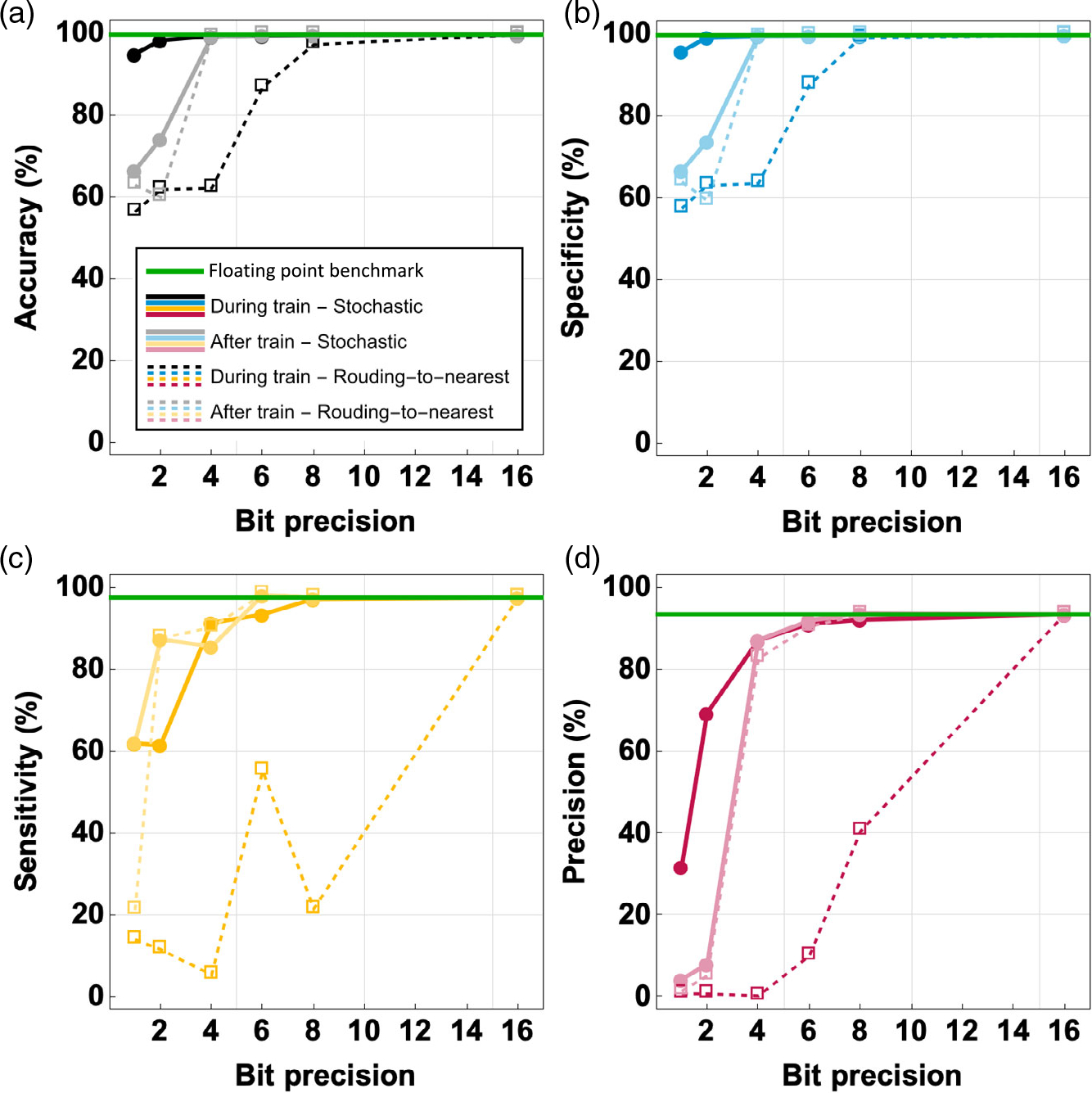
Impact of template bit precision on testing performance: a) accuracy; b) specificity; c) sensitivity; and d) precision for different template quantization levels using rounding-to-nearest method versus using stochastic rounding method during training and after training. The hyperparameters used for all the results in this figure are based on the training results using LR = 0.1, random template initialization, number of neighbors = 8, and binarization after the grayscale training at convergence. Rounding methods are applied during or after training, 800 images were used for training and 200 images for testing.

While the training and testing results are promising using the proposed method, floating-bit precision of the template values numbers is hard to achieve using neuromorphic hardware for inference at the edge due to power, area, and energy constraints. To make the algorithm mappable to hardware, the impact of quantization of the template update has been incorporated in the simulation. Bit precision from 1-bit to 16-bit has been explored in the context of two different types of rounding. The default type of rounding scheme implemented in existing floating-point computers is rounding-to-nearest, ties to even. An alternative method is stochastic rounding which can be particularly useful in network training with low-bit precision arithmetic as a real template value is rounded probabilistically between the lower- and upper-level bounds with a zero-average error and an expected average result of the real template value itself. Each of these rounding methods can be applied on the templates either during or at the end of the training. Figure 8 shows that the overall metrics are high at high-bit precision and approach the nonquantized floating point benchmark as expected. At low-bit precision, the performance decreases depending on the applied rounding method. The lowest performing solution is rounding-to-nearest applied during training. Considering 6-bit fixed-point templates, this method shows accuracy and specificity of around 87%, more than 10 percentage point drop, while the sensitivity dropped to 55% and the precision close to 10%. In comparison, stochastic rounding during training at the same bit precision has accuracy and specificity above 99.6%, a sensitivity of 93.5% and a precision of 91.2%, and only a 1–2 percentage point drop from the floating point benchmark. This is because using this stochastic rounding method, some of the sub-bit information that is discarded by a deterministic rounding scheme can be maintained. The rounding after training has worse performance, but retraining can help achieve higher performance, similar to stochastic rounding during training. Therefore, based on the performance shown in Figure 8, we decided to use the stochastic rounding method for the remainder of the analysis. In addition, 6-bit precision was chosen in line with the state-of-the-art memristor characteristics.^[21,33]^ Other emergent device technologies could be used, but at this time redox memristor devices show the best analog precision, as shown in the study by Wang et al.^[33]^

### 3.2. Inference Robustness

#### 3.2.1. Template Read Variation and Error Rate

In this section, we investigate the potential impact of template read noise on cellular neural networks considering 6-bit cells. For an inference solution, the template retention plays a key role. While analog memory devices can provide a compact, energy-efficient, and even biocompatible solution, they can suffer from read noise and device yield issues. The noise levels in the literature^[34]^ for oxide-based memristive devices indicate typical values of standard deviation of ≈0.007–0.1 due to Johnson–Nyquist noise, random telegraph noise, etc. To achieve high signal-to-noise ratio for read operation, templates with limited bit precision have to be used. Prior work has shown that inference accelerators for perceptron-based neural networks are somewhat immune to partial overlap among neighboring conductance states.^[35–37]^

**Figure 9.**
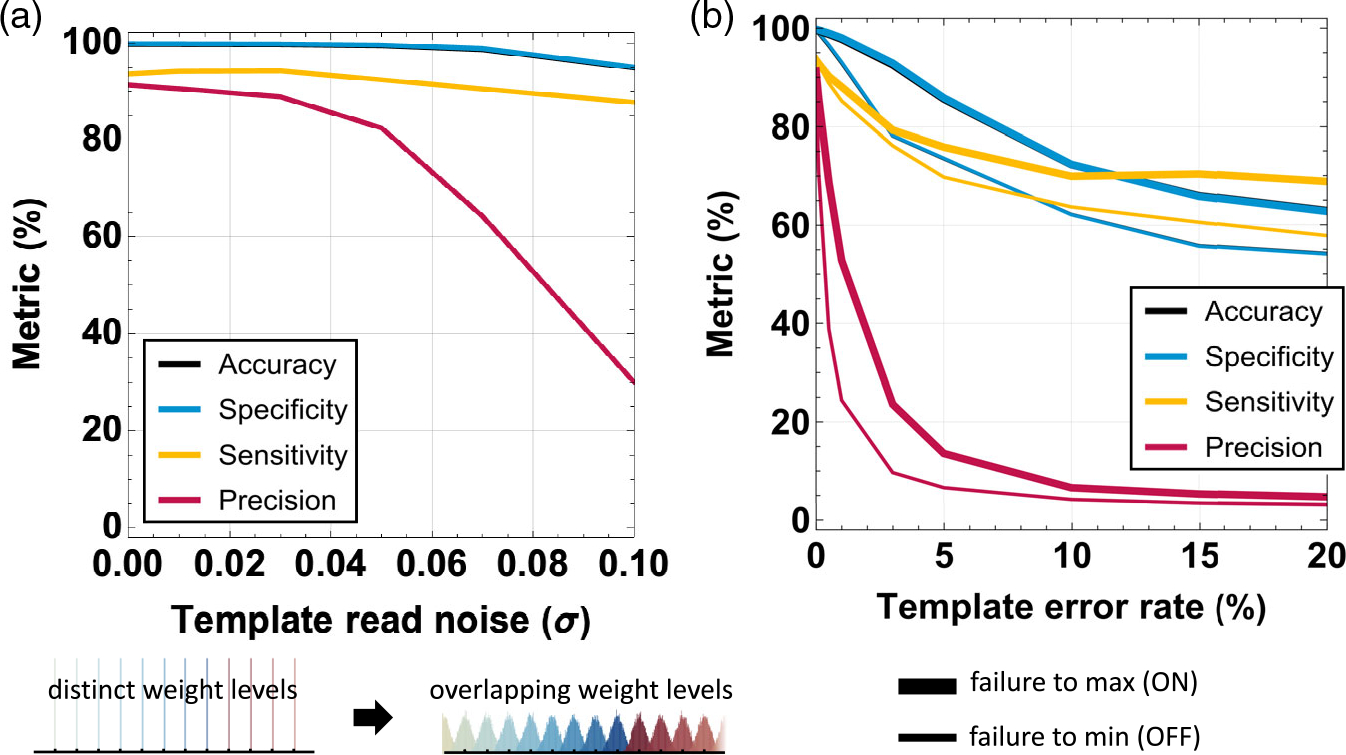
Impact of template read noise and yield on inference performance: a) Read Gaussian noise impacts the metrics, particularly sensitivity and precision, at high levels, when the template values can overlap; b) template yield has a very significant impact, even 1% of values stuck to minimum (OFF) or maximum range (ON) negatively affecting all the metrics. The hyperparameters used for all the results in this figure are based on the training results with template bit precision = 6 bit using LR = 0.1, random template initialization, number of neighbors = 8, and binarization after the grayscale training at convergence. 800 images were used for training and 200 images for testing.

The results in Figure 9a indicate that the performance metrics of the investigated cellular neural networks are also robust to moderate levels of read noise. The noise was modeled as a Gaussian distribution with mean 0 and desired standard deviation, keeping the same value constant for the different levels. While the Johnson–Nyquist noise varies with the conductance level, its value is very small. Other sources of noise, for example, random telegraph noise, can play a much larger role independent of the value of the conductance. This approach is in line with experimental results.^[34]^ For this read noise in the templates, the inference accuracy and specificity of the network are affected minimally. However, the precision starts dropping below 82.44% for read noise above *σ* = 0.05, reaching 6.16% for read noise of *σ* = 0.2.

In comparison, the transfer of model’s obtained template values onto the conductances of analog memory devices requires very high device yield, close to 100%. The inference on a cellular neural network is not robust to failed devices, either the ON or OFF state, with precision dropping below 60% even for 1% of devices being stuck. If the location of the failed devices is known, these fails can be incorporated into the training process. Inference on deep neural networks, such as AlexNet, can tolerate 3% of device fails if the device fail map is considered.^[37]^ However, given the critical requirements for the highest-performing components embedded in a medical implant, this approach is not considered in this work. Instead, having individual chiplets individually tested, selected, and assembled in the proposed distributed network of cellular neural unit can provide a more reliable route for robust integration with the sensors and actuators.

#### 3.2.2. Input Noise

**Figure 10.**
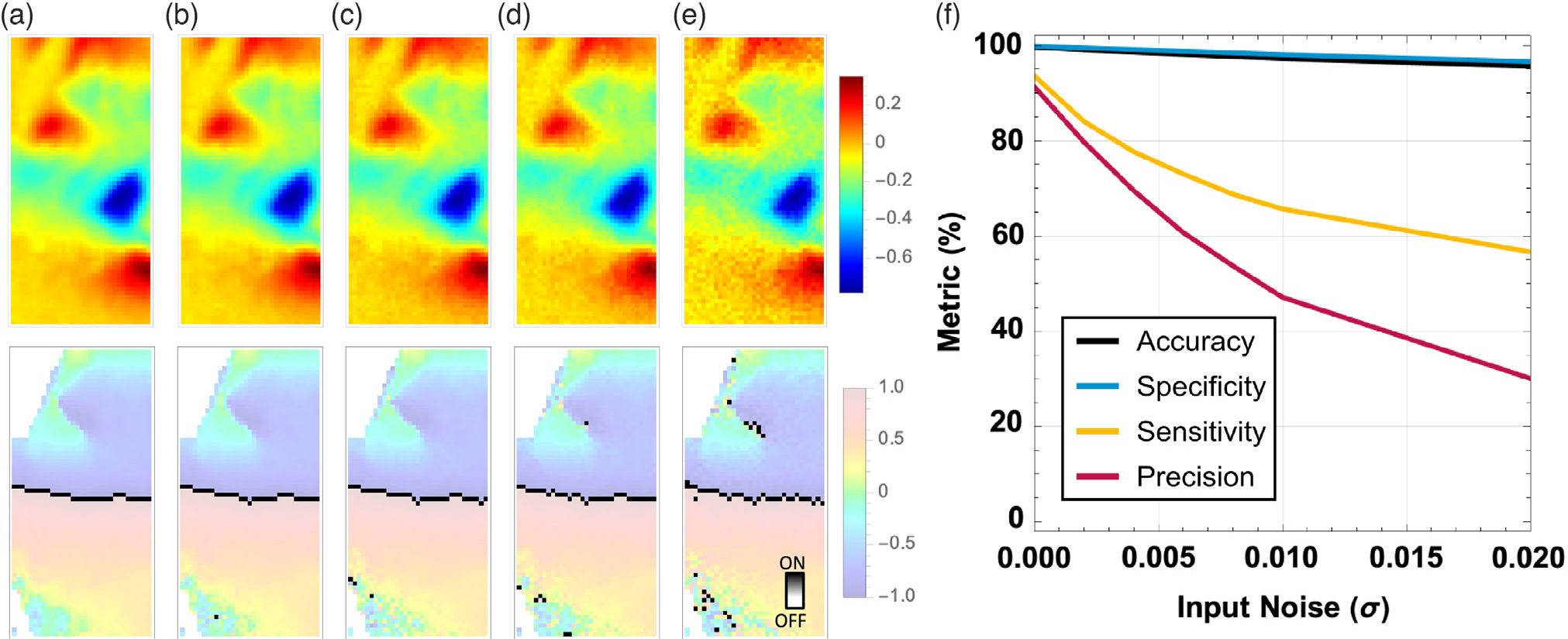
Impact of input noise on inference performance: a) input optical mapping image and its respective Hilbert transform for a) no noise; b) with Gaussian noise *σ* = 0.004; and c) with *σ* = 0.008; d) with *σ* = 0.01 and e) with *σ* = 0.02; f) performance metrics of inference versus the level of noise on the input optical mapping images in the test dataset. The hyperparameters used for all the results in this figure are based on the training results with template bit precision = 6 bit using LR = 0.1, random template initialization, number of neighbors = 8, and binarization after the grayscale training at convergence. 800 images were used for training and 200 images for testing.

Typical electrocardiogram signals from external leads contain many types of noises, baseline wander, powerline interference, electromyographic (EMG) noise, electrode motion artifact noise, etc.^[38,39]^ In an organ-conformal system, some of these sources of noise can be avoided, for example, the powerline interference (50 or 60 Hz noise from mains supply). Other sources of noise can be mitigated using proper signal conditioning within chiplets. A complete system for computing at the edge would contain the necessary preamplification and noise-filtering blocks in the analog front end. Nevertheless, the input in a practical system would have to be based on sensors that measure the local cardiac activity electrically, as shown in Figure 3. Therefore, a source of noise remains the imperfect contact of the electrode array to the heart. In Figure 10, this unwanted behavior is modeled as a Gaussian noise of various standard deviations, applied onto the ideal raw signal before the Hilbert transform step. The results show that the cellular neural network has limited robustness to this noise. As shown in Figure 10c, with input with root mean square (rms or *σ*) Gaussian noise equal with 0.01, the accuracy and specificity of the network are affected minimally, only decreased by ≈4 percentage points, yet the sensitivity and precision of the network are affected quite dramatically, dropping to 65.59% and 39.08% respectively. The network tolerates a noise with *σ* = 0.002, which provides a desirable lower limit of performance to the sensor fabric interfacing with the computing circuitry.

#### 3.2.3. Impact of Neighborhood Size

The results so far have been shown for a neighborhood size of 8. However, it is important to consider the smallest possible neighborhood to reduce overhead in a future hardware implementation. A neighborhood size of 4 was investigated and compared with the 8-neighbor scenario. The results of these investigations are summarized in Table 3. With 4 neighbors, the overall performance is similar with 8 neighbors, within ±1–2 percentage points. The results for testing also show a similar overall performance. For a cellular neural network unit with only four neighbors, the precision decreased by <3 percentage points, but accuracy, specificity, and sensitivity are only affected minimally. The results with different inference nonidealities show similar behavior. Batch training can help overcome these nonidealities if they are incorporated in the models during the training process.

## 4. Discussion

The results show promise for potential implementation of the proposed algorithm into distributed computing hardware for organ-conformal medical implants for high resolution for diagnostic and therapy. In ideal conditions without noise and hardware limitations, the algorithm has high inference performance with accuracy and specificity above 99.7% and with sensitivity of 97.65% and precision 93.83%.

**Figure 11.**
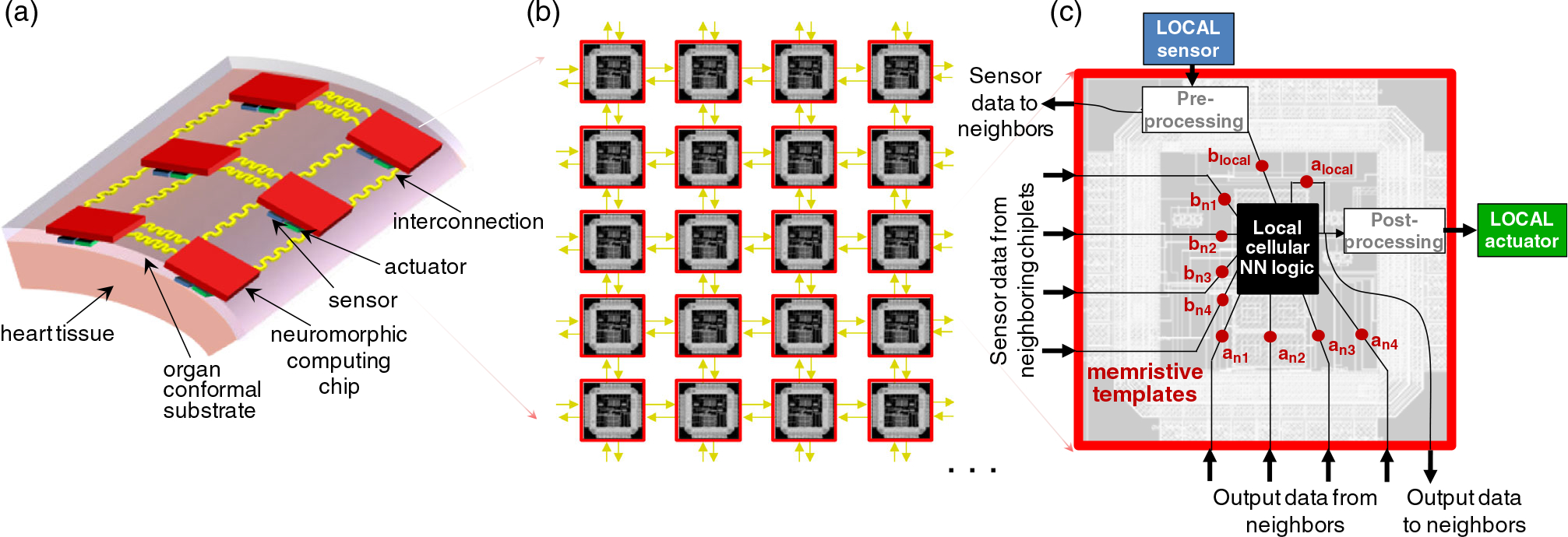
Proposed distributed hardware implementation: a) organ-conformal platform combining sensing, computing and actuation; b) distributed network of neuromorphic computing chips with interconnections that enable the inputs and outputs signals to be shared between neighbors; c) schematic of the functionality of a neuromorphic chip that has a core local cellular neural network-state circuit which determines the output to the actuator based on a set of programmable templates, the inputs from the local and neighboring sensors and the outputs of the local and neighboring chip.

However other constraints have to be considered for practical implementation, such as the hardware architecture, suitability for integration with sensors and actuators, speed, small size and energy efficiency, etc. Traditionally, cellular neural network topologies have been implemented in VLSI technology, particularly for image processing in early 90s^[40]^ and more recently for real-time signal processing at high precision in <ms/frame in applications such as integrated sensing, autonomous vehicles, and mobile robots.^[41–43]^ Recently, few papers have explored the implementation of such cellular topologies using nonvolatile memory devices which promise better performance and energy consumption.^[44,45]^ A hybrid CMOS/memristor implementation was proposed for a standard cellular processing unit where the memristor matrix performs the weight-and-sum operation in the analog domain.^[46]^ The simulation results estimate an area of a processing unit of ≈10 × 10 μm^2^ in the 45 nm mode and an inference speed of 50 ns, showing the feasibility of our real-time compact computing approach. However, all prior work has focused on implementing the entire cellular neural network on a single chip. This is challenging for critical biomedical applications as the yield of emergent devices can be low. In this work, we propose to take advantage of natural organization of a cellular neural network to easily map it to a distributed network of chiplets. As shown in Figure 11a, each computing chiplet could be integrated with one or more sensors and actuators, all embedded in an organ-conformal substrate. For heterogeneous implementation, the cellular neural network logic can be implemented in CMOS, with programmable templates using memristor devices or other programmable nonvolatile memory technologies. Memristors have also shown high retention, extrapolated to hundreds of years,^[47]^ which is needed for long-term use of these implants. The chiplets exchange input and output information for the cellular neural network processing via a fabric of interconnects based on the neighborhood size, as shown in Figure 11b. Each chiplet implements a cell unit of the cellular neural network (Figure 11c), processing only local information from itself and its neighbors, with small size and energy requirements. A chiplet would contain multiple blocks for preprocessing, filtering, amplification, and neural network processing for wavefront detection as well as circuitry for actuation. For neural network processing, the memristor devices can be used for compact nonvolatile storage of the template values, while other blocks can be analog CMOS circuits with fewer transistors and less energy consumption compared with digital equivalents. After synaptic weighting between the templates and the preprocessed inputs, the next block would use this information to determine the X state, by mapping Equation (3) to hardware via *V*–*I* converters and a current summator. Finally, the output block can be implemented, for example, using voltage-controlled voltage source mapping in hardware; Equation (2) with sharp characteristics binarizes the output needed for actuator triggering. For a compact and ultralow-power implementation, an advanced CMOS node (e.g., 22 nm) might be needed to meet the area and power constraints of the chiplet, but the impact of the processing variations on the analog performance would have to be carefully considered. A prototype will be explored in future work as the memristor technology becomes available in more advanced nodes. The network can then be assembled after the templates are transferred to each chiplet by programming the memristive devices. Each chiplet is tested independently, keeping only the chiplets with 100% yield of working memristors.

Practical constraints such as limited bit precision noisy templates and input noise can impact negatively the inference performance. For example, redox memristor devices have shown up to ≈6 bit performance, while other nonvolatile devices are below 4 bit precision.^[33]^ Multiple memristors per template weight can be used, in standalone devices or small arrays.

Nevertheless, hardware-aware training with template limitations, stochastic rounding, and a suitable distributed architecture can maintain inference accuracy and specificity above 99.6% with sensitivity and precision above 90.5%. To save on hardware overhead and make the potential implementation even more compact and energy efficient, the neighborhood can be reduced to four neighbors, each cell having connections only to other cells immediately above, below, left, and right. This approach comes at a tolerable performance penalty, of only 2–3% for the most critical metrics, sensitivity, and precision.

Future work will focus on the challenges related to practical implementation, particularly at scale. The next steps are to develop a chiplet design that implements the proposed algorithm and has suitable metrics for the cardiac application at hand: 1) fast response time for sensor input preprocessing, cellular neural network computing, and postprocessing to actuate the therapy, enabling decision-making in a few milliseconds for real-time operation; 2) small chip footprint, enabling unobtrusive integration onto the organ conformal platform without affecting the cardiac tissue in vivo. An initial estimate is 100–500 μm maximum lateral dimensions, so advanced CMOS nodes would likely have to be used; and 3) high data throughput, enabling data processing from thousands of sensors. Studies are ongoing to determine the minimum density needed to detect wavefronts, but an initial estimate is ≈ 10^3^–10^4^ sensors; d) ultralow power vital for implantable biomedical technologies that are powered by energy-harvesting systems that deliver μWs.^[48]^

Another focus of future work will be expanding the dataset of cardiac signals, from both optical and electrical mapping. Abundant data are typically needed for training neural networks with high performance and this work has shown that cellular neural networks are no exception. A large scale dataset with labeled spatiotemporal cardiac recordings and respective wavefronts labeled will speed up the development of new computing algorithms for classification of wavefronts (and applicable other similar spatiotemporal biorecordings) faster, more reliably, and more efficiently. The challenge is that in the case of cardiac maps, this data can be difficult to obtain ex vivo for across the entire ventricular tissue in human hearts. As donor hearts are particularly challenging to source for research studies, an alternative is to develop an extensive datasets for rabbit, pig, or dog hearts. Both sexes and various ages would have to be incorporated to capture any specific differences in electrophysiological parameters.^[49–52]^

## 5. Conclusion

This article proposes a compact and efficient computing solution based on cellular neural networks to close the data loop in organ conformal devices in an efficient manner. On-going scientific and clinical research is needed to understand the spatiotemporal complexity of wave propagation in arrhythmias. Advanced engineering solutions are required for this work, with innovative computing technologies being crucial due to difficult real-time data processing constraints. The proposed algorithm has shown promising performance across all investigated metrics and is mappable to a distributed hardware implementation based on chiplets, suitable for integration with sensors and actuators in a dense fashion. Memristor devices can be used to store the template values and provide a compact design. Future work will focus on achieving a suitable circuit design for the proposed chiplet that meets the practical constraints regarding area, speed, and energy consumption. An integrated system with high-resolution sensing, high-performance computing, and low-energy actuation, capable of millisecond response times, will enable ground-breaking technologies, like ultrafast detection and therapy for heart diseases. Moreover, bioelectric signals govern the functionality of a number of vital human organs, like the brain, heart, muscles, gut, etc., so the proposed solution could find applications in the detection of other bioelectric anomalies that require high definition for smart diagnostics and real-time therapy.

## Figures and Tables

**Table 1.** Performance matrix.

		Desired output pixel	

Obtained output pixel		ON	OFF
	ON	True Positive (TP)	False Positive (FP)
	OFF	False Negative (FN)	True Negative (TN)

**Table 2.** Initialization templates used for a system with eight neighbors.

		Zero template			Example of random template			Edge detection template	

Template A	0	0	0	−0.6849	0.3077	0.189	0	0	0
	0	0	0	−0.1537	0.5972	0.5028	0	0	0
	0	0	0	−0.1185	−0.3394	−0.3372	0	0	0
Template B	0	0	0	−0.4339	−0.4386	−0.0429	−1	−1	−1
	0	0	0	−0.9216	−0.6144	−0.7459	−1	8	−1
	0	0	0	−0.8378	0.014	−0.2083	−1	−1	−1
Bias I		0			0.777			1	

**Table 3.** Neighborhood comparison for best accuracy results. Results obtained on full training and testing sets, using a learning rate of 1.0, binarization after training, and a batch size of 128 the synaptic templates feature 0.5% variability and 4-bit precision and are limited to ±1 normalized values.

	8 neighbors		4 neighbors	
	Training	Testing	Training	Testing

Accuracy	99.21%	99.16%	99.18%	99.13%
Specificity	99.86%	99.79%	99.89%	99.73%
Sensitivity	76.91%	76.46%	74.64%	77.40%
Precision	92.64%	90.15%	93.99%	87.50%

## Data Availability

The data that support the findings of this study are available from the corresponding author upon reasonable request.

## Appendix A.

Detailed Algorithm Description
